# Reviewer acknowledgement 2013

**DOI:** 10.1186/2052-1839-14-1

**Published:** 2014-01-15

**Authors:** Peter O'Donovan

**Affiliations:** 1 BioMed Central, 236 Gray’s Inn, Road, London, WC1X 8HB, UK

## Abstract

**Contributing reviewers:**

The editors of *BMC Hematology* would like to thank all our reviewers who have contributed to the journal in Volume 13 (2013).

## 

Anil Agarwal

USA

Marly Cardoso

Brazil

Emre Cecen

Turkey

Romanee Chaiwarith

Thailand

Sudipta Chakrabarti

India

Cheng-Shyong Chang

Taiwan

Hung Chang

Taiwan

Ying-Jun Chang

China

Ploenchan Chetchotisakd

Timor-Leste

Alessia Colosimo

Italy

Anna Czyz

Poland

Srdjan Denic

United Arab Emirates

Jean Donadieu

France

Amol Dongre

India

Nikolaos Drakoulis

Greece

Youssef Farag

USA

Lucia Farina

Italy

Guido Finazzi

Italy

Ivan Gentile

Italy

Vineeta Gupta

India

Takakazu Higuchi

Japan

Johannes Hoffmann

Germany

Brian Jackson

USA

Theo Kim

Germany

Thomas Lecompte

Switzerland

Heather Leitch

Canada

Hassan Mansouritorghabeh

Iran

Saskia Middeldorp

Netherlands

Dragana Milojkovic

UK

Satu Mustjoki

Finland

Ravi Nistala

USA

Adam Olszewski

USA

William Kwame Boakye Ansah Owiredu

Ghana

Anthony Oyekunle

Nigeria

Srdjan Pasic

Serbia

Anette Pedersen

Denmark

Christopher Pell

Netherlands

Linda Pickle

USA

Marcin Protasiewicz

Poland

Zohreh Rahimi

Iran

Kishor Raja

UK

Emily Rousham

UK

Deborah Rund

Israel

Takayuki Saitoh

Japan

Malabika Sarker

Bangladesh

Zonghong Shao

China

Yoko Shibata

Japan

Patrick Sullivan

USA

Junko Tanaka

Japan

James Taylor

USA

Recep Tekin

Turkey

Hugo ten Cate

Netherlands

Mahmoud Torabi

Canada

Thomas Uldrick

USA

Inge Verbrugge

Netherlands

Bert Verbruggen

Netherlands

Fiona Walter

UK

Jeffrey Weitz

Canada

C. Cameron Yin

USA

Sirous Zeinali

Iran

## Pre-publication history

The pre-publication history for this paper can be accessed here:

http://www.biomedcentral.com/2052-1839/14/1/prepub

